# Supplementary material for: Development of a PCR-based, genetic marker resource for the tomato-like nightshade relative, Solanum lycopersicoides using whole genome sequence analysis
Source: PLoS One. 2020 Nov 23;15(11):e0242882. doi: 10.1371/journal.pone.0242882 (PMC7682897; doi:10.1371/journal.pone.0242882)
Supplement: S1 Table — (DOCX) [file pone.0242882.s001.docx]

**S1 Table. *S.* *lycopersicoides*-specific DNA markers developed using whole genome sequence analysis*.***

| No. | Primer Name | Source species | Marker type | Repeat motif | Expected amplicon size (bp) | Chr^e^ | Map position^f^ (Mb) |
| --- | --- | --- | --- | --- | --- | --- | --- |
| 1 | SLM^a^ 01 | *S. lycopersicoides* | SSR^c^ | AC^11^ | 251 | 1 | 0.38 |
| 2 | SLM 08 | *S. lycopersicoides* | SSR | AATG^5^ | 267 | 1 | 9.30 |
| 3 | SLM 09 | *S. lycopersicoides* | SSR | TTG^7^ | 267 | 1 | 13.80 |
| 4 | SLM 10 | *S. lycopersicoides* | SSR | AT^17^ | 245 | 1 | 17.80 |
| 5 | SLM 11 | *S. lycopersicoides* | SSR | AT^13^ | 211 | 1 | 36.50 |
| 6 | SLM 12 | *S. lycopersicoides* | SSR | TA^12^ | 320 | 1 | 62.70 |
| 7 | SLM 05 | *S. lycopersicoides* | SSR | AT^20^ | 195 | 1 | 72.51 |
| 8 | SLM 06 | *S. lycopersicoides* | SSR | TAT^15^ | 219 | 1 | 77.21 |
| 9 | SLM 07 | *S. lycopersicoides* | SSR | TTCCTC^5^ | 341 | 1 | 81.18 |
| 10 | SLM 13 | *S. lycopersicoides* | SSR | AAAT^5^ | 381 | 2 | 21.60 |
| 11 | SLM 14 | *S. lycopersicoides* | SSR | TA^14^ | 217 | 2 | 30.50 |
| 12 | SLM 15 | *S. lycopersicoides* | SSR | TAA^7^ | 223 | 2 | 39.10 |
| 13 | SLM 16 | *S. lycopersicoides* | SSR | CCTCTC^5^ | 224 | 2 | 45.10 |
| 14 | SLM 17 | *S. lycopersicoides* | SSR | TA^12^ | 217 | 2 | 51.00 |
| 15 | SLM 26 | *S. lycopersicoides* | SSR | AAAGA^5^ | 245 | 3 | - ^g^ |
| 16 | SLM 18 | *S. lycopersicoides* | SSR | TA^10^ | 248 | 3 | 9.38 |
| 17 | SLM 181 | *S. lycopersicoides* | SSR | TTGTAA^6^ | 214 | 3 | 16.22 |
| 18 | SLM 19 | *S. lycopersicoides* | SSR | TTC^7^ | 288 | 3 | 21.10 |
| 19 | SLM 20 | *S. lycopersicoides* | SSR | TA^17^ | 274 | 3 | 27.20 |
| 20 | SLM 21 | *S. lycopersicoides* | SSR | AGAA^5^ | 293 | 3 | 33.33 |
| 21 | SLM 182 | *S. lycopersicoides* | SSR | TA^9^ | 295 | 3 | 41.01 |
| 22 | SLM 183 | *S. lycopersicoides* | SSR | AT^8^ | 257 | 3 | 46.34 |
| 23 | SLM 22 | *S. lycopersicoides* | SSR | TTTACA^6^ | 205 | 3 | 48.66 |
| 24 | SLM 23 | *S. lycopersicoides* | SSR | CT^13^ | 336 | 3 | 54.40 |
| 25 | SLM 184 | *S. lycopersicoides* | SSR | TA^15^ | 102 | 3 | 57.51 |
| 26 | SLM 24 | *S. lycopersicoides* | SSR | AG^10^ | 280 | 3 | 59.87 |
| 27 | SLM 185 | *S. lycopersicoides* | SSR | GGGGTG^6^ | 199 | 3 | 63.03 |
| 28 | SLM 25 | *S. lycopersicoides* | SSR | TA^13^ | 208 | 3 | 65.60 |
| 29 | SLM 186 | *S. lycopersicoides* | SSR | TA^16^ | 337 | 3 | 69.46 |
| 30 | SLM 34 | *S. lycopersicoides* | SSR | AT^18^ | 268 | 4 | - |
| 31 | SLM 36 | *S. lycopersicoides* | SSR | TAT^12^ | 268 | 4 | - |
| 32 | SLM 37 | *S. lycopersicoides* | SSR | CTT^7^ | 230 | 4 | - |
| 33 | SLM 27 | *S. lycopersicoides* | SSR | AAAT^5^ | 233 | 4 | 3.87 |
| 34 | SLM 28 | *S. lycopersicoides* | SSR | TA^10^ | 200 | 4 | 9.57 |
| 35 | SLM 29 | *S. lycopersicoides* | SSR | AT^12^ | 214 | 4 | 15.70 |
| 36 | SLM 187 | *S. lycopersicoides* | SSR | TA^5^ | 202 | 4 | 21.19 |
| 37 | SLM 30 | *S. lycopersicoides* | SSR | AAGA^7^ | 236 | 4 | 27.38 |
| 38 | SLM 31 | *S. lycopersicoides* | SSR | GGCATC^5^ | 253 | 4 | 33.26 |
| 39 | SLM 188 | *S. lycopersicoides* | SSR | AT^11^ | 241 | 4 | 35.34 |
| 40 | SLM 189 | *S. lycopersicoides* | SSR | TCT^5^ | 161 | 4 | 38.25 |
| 41 | SLM 32 | *S. lycopersicoides* | SSR | TTTA^7^ | 276 | 4 | 39.00 |
| 42 | SLM 190 | *S. lycopersicoides* | SSR | TA^24^ | 191 | 4 | 40.03 |
| 43 | SLM 33 | *S. lycopersicoides* | SSR | TA^11^ | 303 | 4 | 44.80 |
| 44 | SLM 191 | *S. lycopersicoides* | SSR | AG^9^ | 212 | 4 | 50.45 |
| 45 | SLM 192 | *S. lycopersicoides* | SSR | TA^10^ | 227 | 4 | 53.44 |
| 46 | SLM 35 | *S. lycopersicoides* | SSR | TAT^11^ | 259 | 4 | 56.09 |
| 47 | SLM 193 | *S. lycopersicoides* | SSR | AT^11^ | 260 | 4 | 58.23 |
| 48 | SLM 194 | *S. lycopersicoides* | SSR | TA^7^ | 220 | 4 | 60.19 |
| 49 | SLM 195 | *S. lycopersicoides* | SSR | TA^10^ | 176 | 4 | 63.01 |
| 50 | SLM 196 | *S. lycopersicoides* | SSR | AG^12^ | 181 | 4 | 66.29 |
| 51 | SLM 38 | *S. lycopersicoides* | SSR | AG^10^ | 266 | 5 | - |
| 52 | SLM 41 | *S. lycopersicoides* | SSR | GAAA^5^ | 325 | 5 | - |
| 53 | SLM 42 | *S. lycopersicoides* | SSR | TCA^7^ | 277 | 5 | - |
| 54 | SLM 45 | *S. lycopersicoides* | SSR | TA^16^ | 200 | 5 | - |
| 55 | SLM 46 | *S. lycopersicoides* | SSR | AT^15^ | 333 | 5 | - |
| 56 | SLM 47 | *S. lycopersicoides* | SSR | TATAT^7^ | 219 | 5 | - |
| 57 | SLM 48 | *S. lycopersicoides* | SSR | AAT^8^ | 261 | 5 | - |
| 58 | SLM 105 | *S. lycopersicoides* | SSR | ATTA^7^ | 366 | 5 | 3.29 |
| 59 | SLM 39 | *S. lycopersicoides* | SSR | AT^14^ | 214 | 5 | 9.59 |
| 60 | SLM 40 | *S. lycopersicoides* | SSR | GGGGTG^5^ | 263 | 5 | 15.26 |
| 61 | SLM 106 | *S. lycopersicoides* | SSR | ATATT^5^ | 201 | 5 | 27.16 |
| 62 | SLM 43 | *S. lycopersicoides* | SSR | AT^22^ | 371 | 5 | 32.97 |
| 63 | SLM 44 | *S. lycopersicoides* | SSR | TGG^7^ | 216 | 5 | 39.72 |
| 64 | SLM 107 | *S. lycopersicoides* | SSR | TC^9^ | 273 | 5 | 39.72 |
| 65 | SLM 108 | *S. lycopersicoides* | SSR | TAA^19^ | 382 | 5 | 55.32 |
| 66 | SLM 109 | *S. lycopersicoides* | SSR | TA^17^ | 288 | 5 | 60.09 |
| 67 | SLM 110 | *S. lycopersicoides* | SSR | TTG^6^ | 248 | 5 | 65.72 |
| 68 | SLM 50 | *S. lycopersicoides* | SSR | AT^14^ | 255 | 6 | - |
| 69 | SLM 56 | *S. lycopersicoides* | SSR | TC^12^ | 241 | 6 | - |
| 70 | SLM 49 | *S. lycopersicoides* | SSR | TA^18^ | 258 | 6 | 0.01 |
| 71 | SLM 111 | *S. lycopersicoides* | SSR | CT^10^ | 255 | 6 | 17.07 |
| 72 | SLM 51 | *S. lycopersicoides* | SSR | TTAA^6^ | 254 | 6 | 19.70 |
| 73 | SLM 112 | *S. lycopersicoides* | SSR | CT^9^ | 207 | 6 | 27.60 |
| 74 | SLM 52 | *S. lycopersicoides* | SSR | TAAT^5^ | 316 | 6 | 27.84 |
| 75 | SLM 113 | *S. lycopersicoides* | SSR | AG^9^ | 248 | 6 | 33.42 |
| 76 | SLM 53 | *S. lycopersicoides* | SSR | TA^23^ | 273 | 6 | 33.59 |
| 77 | SLM 54 | *S. lycopersicoides* | SSR | AT^11^ | 201 | 6 | 39.00 |
| 78 | SLM 55 | *S. lycopersicoides* | SSR | TGT^7^ | 269 | 6 | 45.07 |
| 79 | SLM 58 | *S. lycopersicoides* | SSR | TGA^8^ | 204 | 7 | - |
| 80 | SLM 59 | *S. lycopersicoides* | SSR | AAT^10^ | 355 | 7 | - |
| 81 | SLM 64 | *S. lycopersicoides* | SSR | AT^18^ | 257 | 7 | - |
| 82 | SLM 65 | *S. lycopersicoides* | SSR | AT^16^ | 216 | 7 | - |
| 83 | SLM 57 | *S. lycopersicoides* | SSR | ACT^7^ | 230 | 7 | 0.04 |
| 84 | SLM 114 | *S. lycopersicoides* | SSR | AT^9^ | 363 | 7 | 6.43 |
| 85 | SLM 60 | *S. lycopersicoides* | SSR | TA^11^ | 377 | 7 | 18.88 |
| 86 | SLM 61 | *S. lycopersicoides* | SSR | TAT^13^ | 309 | 7 | 24.90 |
| 87 | SLM 63 | *S. lycopersicoides* | SSR | CT^14^ | 283 | 7 | 35.38 |
| 88 | SLM 62 | *S. lycopersicoides* | SSR | AAGA^5^ | 229 | 7 | 36.74 |
| 89 | SLM 173 | *S. lycopersicoides* | SSR | AC^6^ | 288 | 7 | 45.34 |
| 90 | SLM 174 | *S. lycopersicoides* | SSR | CCCTGT^5^ | 171 | 7 | 47.72 |
| 91 | SLM 175 | *S. lycopersicoides* | SSR | TGA^6^ | 245 | 7 | 49.05 |
| 92 | SLM 176 | *S. lycopersicoides* | SSR | AT^7^ | 290 | 7 | 51.18 |
| 93 | SLM 66 | *S. lycopersicoides* | SSR | AT^10^ | 218 | 7 | 51.49 |
| 94 | SLM 177 | *S. lycopersicoides* | SSR | AT^8^ | 223 | 7 | 54.63 |
| 95 | SLM 67 | *S. lycopersicoides* | SSR | AT^15^ | 278 | 7 | 57.49 |
| 96 | SLM 178 | *S. lycopersicoides* | SSR | ACT^5^ | 177 | 7 | 60.36 |
| 97 | SLM 68 | *S. lycopersicoides* | SSR | TTC^9^ | 328 | 7 | 62.99 |
| 98 | SLM 179 | *S. lycopersicoides* | SSR | TAT^8^ | 233 | 7 | 63.73 |
| 99 | SLM 180 | *S. lycopersicoides* | SSR | GAA^5^ | 290 | 7 | 66.07 |
| 100 | SLM 69 | *S. lycopersicoides* | SSR | AT^10^ | 294 | 7 | 68.00 |
| 101 | SLM 70 | *S. lycopersicoides* | SSR | TTTAG^5^ | 209 | 8 | 0.02 |
| 102 | SLM 71 | *S. lycopersicoides* | SSR | TG^11^ | 279 | 8 | 6.50 |
| 103 | SLM 72 | *S. lycopersicoides* | SSR | TA^13^ | 294 | 8 | 12.87 |
| 104 | SLM 115 | *S. lycopersicoides* | SSR | AT^10^ | 237 | 8 | 14.64 |
| 105 | SLM 116 | *S. lycopersicoides* | SSR | GGA^8^ | 366 | 8 | 16.14 |
| 106 | SLM 117 | *S. lycopersicoides* | SSR | AT^24^ | 333 | 8 | 22.88 |
| 107 | SLM 118 | *S. lycopersicoides* | SSR | AT^11^ | 240 | 8 | 30.14 |
| 108 | SLM 73 | *S. lycopersicoides* | SSR | CCCCG^5^ | 263 | 8 | 30.30 |
| 109 | SLM 119 | *S. lycopersicoides* | SSR | TTA^15^ | 327 | 8 | 33.72 |
| 110 | SLM 74 | *S. lycopersicoides* | SSR | CCA^7^ | 236 | 8 | 36.30 |
| 111 | SLM 120 | *S. lycopersicoides* | SSR | AAG^10^ | 201 | 8 | 45.61 |
| 112 | SLM 75 | *S. lycopersicoides* | SSR | AT^10^ | 264 | 8 | 46.61 |
| 113 | SLM 121 | *S. lycopersicoides* | SSR | TA^9^ | 234 | 8 | 48.30 |
| 114 | SLM 122 | *S. lycopersicoides* | SSR | AGA^19^ | 323 | 8 | 51.13 |
| 115 | SLM 123 | *S. lycopersicoides* | SSR | AAT^8^ | 200 | 8 | 54.20 |
| 116 | SLM 76 | *S. lycopersicoides* | SSR | AG^10^ | 311 | 8 | 54.52 |
| 117 | SLM 124 | *S. lycopersicoides* | SSR | TA^24^ | 309 | 8 | 56.88 |
| 118 | SLM 125 | *S. lycopersicoides* | SSR | AAC^7^ | 279 | 8 | 59.59 |
| 119 | SLM 77 | *S. lycopersicoides* | SSR | CAA^7^ | 271 | 8 | 59.84 |
| 120 | SLM 126 | *S. lycopersicoides* | SSR | AT^9^ | 338 | 8 | 62.87 |
| 121 | SLM 78 | *S. lycopersicoides* | SSR | TC^16^ | 216 | 8 | 65.86 |
| 122 | SLM 85 | *S. lycopersicoides* | SSR | TA^16^ | 201 | 9 | - |
| 123 | SLM 86 | *S. lycopersicoides* | SSR | TAT^7^ | 211 | 9 | - |
| 124 | SLM 88 | *S. lycopersicoides* | SSR | CCT^7^ | 228 | 9 | - |
| 125 | SLM 79 | *S. lycopersicoides* | SSR | AT^13^ | 215 | 9 | 0.01 |
| 126 | SLM 127 | *S. lycopersicoides* | SSR | AG^10^ | 228 | 9 | 0.02 |
| 127 | SLM 80 | *S. lycopersicoides* | SSR | TA^10^ | 202 | 9 | 6.18 |
| 128 | SLM 128 | *S. lycopersicoides* | SSR | TA^10^ | 334 | 9 | 6.89 |
| 129 | SLM 129 | *S. lycopersicoides* | SSR | TA^18^ | 245 | 9 | 9.26 |
| 130 | SLM 130 | *S. lycopersicoides* | SSR | AT^10^ | 317 | 9 | 14.43 |
| 131 | SLM 131 | *S. lycopersicoides* | SSR | AT^14^ | 380 | 9 | 16.67 |
| 132 | SLM 81 | *S. lycopersicoides* | SSR | AACCCT^5^ | 218 | 9 | 24.00 |
| 133 | SLM 132 | *S. lycopersicoides* | SSR | AT^19^ | 322 | 9 | 24.85 |
| 134 | SLM 82 | *S. lycopersicoides* | SSR | TAAT^6^ | 204 | 9 | 30.00 |
| 135 | SLM 133 | *S. lycopersicoides* | SSR | AT^20^ | 345 | 9 | 30.92 |
| 136 | SLM 134 | *S. lycopersicoides* | SSR | AT^13^ | 239 | 9 | 36.89 |
| 137 | SLM 135 | *S. lycopersicoides* | SSR | TGGCTG^6^ | 285 | 9 | 39.76 |
| 138 | SLM 83 | *S. lycopersicoides* | SSR | ATA^8^ | 219 | 9 | 42.26 |
| 139 | SLM 84 | *S. lycopersicoides* | SSR | TATATG^6^ | 208 | 9 | 47.70 |
| 140 | SLM 136 | *S. lycopersicoides* | SSR | AC^7^ | 214 | 9 | 49.42 |
| 141 | SLM 137 | *S. lycopersicoides* | SSR | ATT^6^ | 309 | 9 | 54.46 |
| 142 | SLM 138 | *S. lycopersicoides* | SSR | TA^7^ | 254 | 9 | 57.11 |
| 143 | SLM 139 | *S. lycopersicoides* | SSR | AT^7^ | 313 | 9 | 60.19 |
| 144 | SLM 140 | *S. lycopersicoides* | SSR | AT^19^ | 295 | 9 | 63.80 |
| 145 | SLM 87 | *S. lycopersicoides* | SSR | AAC^7^ | 272 | 9 | 65.49 |
| 146 | SLM 141 | *S. lycopersicoides* | SSR | GA^9^ | 205 | 9 | 66.03 |
| 147 | SLM 142 | *S. lycopersicoides* | SSR | TAA^9^ | 288 | 9 | 69.30 |
| 148 | SLM 143 | *S. lycopersicoides* | SSR | AT^15^ | 221 | 9 | 72.28 |
| 149 | SLM 93 | *S. lycopersicoides* | SSR | TA^12^ | 233 | 10 | - |
| 150 | SLM 144 | *S. lycopersicoides* | SSR | AT^9^ | 317 | 10 | 0.19 |
| 151 | SLM 89 | *S. lycopersicoides* | SSR | TTA^8^ | 235 | 10 | 0.20 |
| 152 | SLM 02 | *S. lycopersicoides* | SSR | AAG^6^ | 239 | 10 | 5.83 |
| 153 | SLM 03 | *S. lycopersicoides* | SSR | TC^5^ | 262 | 10 | 11.27 |
| 154 | SLM 145 | *S. lycopersicoides* | SSR | TA^20^ | 233 | 10 | 13.34 |
| 155 | SLM 90 | *S. lycopersicoides* | SSR | ATT^9^ | 261 | 10 | 13.73 |
| 156 | SLM 146 | *S. lycopersicoides* | SSR | AG^9^ | 218 | 10 | 17.89 |
| 157 | SLM 91 | *S. lycopersicoides* | SSR | TTAT^5^ | 254 | 10 | 19.45 |
| 158 | SLM 147 | *S. lycopersicoides* | SSR | TA^5^ | 222 | 10 | 19.71 |
| 159 | SLM 148 | *S. lycopersicoides* | SSR | TAG^5^ | 231 | 10 | 21.38 |
| 160 | SLM 149 | *S. lycopersicoides* | SSR | TA^10^ | 272 | 10 | 23.92 |
| 161 | SLM 04 | *S. lycopersicoides* | SSR | TC^15^ | 260 | 10 | 26.44 |
| 162 | SLM 150 | *S. lycopersicoides* | SSR | CT^10^ | 232 | 10 | 28.95 |
| 163 | SLM 151 | *S. lycopersicoides* | SSR | ATC^5^ | 310 | 10 | 30.54 |
| 164 | SLM 152 | *S. lycopersicoides* | SSR | TTA^14^ | 231 | 10 | 33.40 |
| 165 | SLM 92 | *S. lycopersicoides* | SSR | TC^13^ | 290 | 10 | 36.50 |
| 166 | SLM 153 | *S. lycopersicoides* | SSR | GA^7^ | 227 | 10 | 39.26 |
| 167 | SLM 154 | *S. lycopersicoides* | SSR | TTC^7^ | 236 | 10 | 42.76 |
| 168 | SLM 155 | *S. lycopersicoides* | SSR | TA^12^ | 239 | 10 | 45.53 |
| 169 | SLM 156 | *S. lycopersicoides* | SSR | AG^8^ | 275 | 10 | 48.08 |
| 170 | SLM 94 | *S. lycopersicoides* | SSR | AAT^11^ | 349 | 10 | 54.42 |
| 171 | SLM 157 | *S. lycopersicoides* | SSR | GAA^12^ | 218 | 10 | 57.44 |
| 172 | SLM 158 | *S. lycopersicoides* | SSR | TCAAGA^5^ | 304 | 10 | 60.45 |
| 173 | SLM 95 | *S. lycopersicoides* | SSR | TATTT^5^ | 270 | 10 | 62.42 |
| 174 | SLM 159 | *S. lycopersicoides* | SSR | CAAGAG^5^ | 236 | 10 | 65.25 |
| 175 | SLM 96 | *S. lycopersicoides* | SSR | CAA^8^ | 206 | 11 | 41.53 |
| 176 | SLM 101 | *S. lycopersicoides* | SSR | TA^23^ | 249 | 12 | - |
| 177 | SLM 102 | *S. lycopersicoides* | SSR | AT^18^ | 236 | 12 | - |
| 178 | SLM 103 | *S. lycopersicoides* | SSR | ATT^7^ | 203 | 12 | - |
| 179 | SLM 104 | *S. lycopersicoides* | SSR | ACA^7^ | 207 | 12 | - |
| 180 | SLM 97 | *S. lycopersicoides* | SSR | ATGT^5^ | 210 | 12 | 18.84 |
| 181 | SLM 98 | *S. lycopersicoides* | SSR | GAG^9^ | 206 | 12 | 28.27 |
| 182 | SLM 172 | *S. lycopersicoides* | SSR | CT^6^ | 276 | 12 | 32.75 |
| 183 | SLM 99 | *S. lycopersicoides* | SSR | TTCT^5^ | 237 | 12 | 33.32 |
| 184 | SLM 163 | *S. lycopersicoides* | SSR | ATT^10^ | 319 | 12 | 37.15 |
| 185 | SLM 164 | *S. lycopersicoides* | SSR | TCT^12^ | 301 | 12 | 39.14 |
| 186 | SLM 165 | *S. lycopersicoides* | SSR | TTATA^8^ | 207 | 12 | 42.55 |
| 187 | SLM 166 | *S. lycopersicoides* | SSR | TA^12^ | 195 | 12 | 45.33 |
| 188 | SLM 167 | *S. lycopersicoides* | SSR | TTA^6^ | 277 | 12 | 48.96 |
| 189 | SLM 168 | *S. lycopersicoides* | SSR | AT^7^ | 132 | 12 | 50.83 |
| 190 | SLM 169 | *S. lycopersicoides* | SSR | ATT^5^ | 262 | 12 | 55.08 |
| 191 | SLM 170 | *S. lycopersicoides* | SSR | TA^15^ | 318 | 12 | 57.31 |
| 192 | SLM 100 | *S. lycopersicoides* | SSR | AT^10^ | 295 | 12 | 58.38 |
| 193 | SLM 171 | *S. lycopersicoides* | SSR | TA^21^ | 196 | 12 | 60.09 |
| 194 | SLM 162 | *S. lycopersicoides* | SSR | AT^15^ | 189 | 12 | 63.64 |
| 195 | SLM 161 | *S. lycopersicoides* | SSR | TG^10^ | 316 | 12 | 65.63 |
| 196 | SLM 160 | *S. lycopersicoides* | SSR | CA^6^ | 360 | 12 | 67.12 |
| 197 | SLYD^b^ 01 | *S. lycopersicoides* | indel^d^ | - | 331 | 1 | 0.04 |
| 198 | SLYD 81 | *S. lycopersicoides* | indel | - | 318 | 1 | 2.74 |
| 199 | SLYD 09 | *S. lycopersicoides* | indel | - | 262 | 1 | 3.03 |
| 200 | SLYD 10 | *S. lycopersicoides* | indel | - | 282 | 1 | 6.17 |
| 201 | SLYD 82 | *S. lycopersicoides* | indel | - | 282 | 1 | 8.63 |
| 202 | SLYD 83 | *S. lycopersicoides* | indel | - | 223 | 1 | 11.69 |
| 203 | SLYD 11 | *S. lycopersicoides* | indel | - | 381 | 1 | 15.50 |
| 204 | SLYD 84 | *S. lycopersicoides* | indel | - | 202 | 1 | 19.84 |
| 205 | SLYD 108 | *S. lycopersicoides* | indel | - | 164 | 1 | 24.34 |
| 206 | SLYD 02 | *S. lycopersicoides* | indel | - | 321 | 1 | 27.85 |
| 207 | SLYD 03 | *S. lycopersicoides* | indel | - | 320 | 1 | 30.76 |
| 208 | SLYD 112 | *S. lycopersicoides* | indel | - | 248 | 1 | 32.26 |
| 209 | SLYD 109 | *S. lycopersicoides* | indel | - | 174 | 1 | 35.37 |
| 210 | SLYD 96 | *S. lycopersicoides* | indel | - | 214 | 1 | 39.81 |
| 211 | SLYD 94 | *S. lycopersicoides* | indel | - | 205 | 1 | 40.99 |
| 212 | SLYD 103 | *S. lycopersicoides* | indel | - | 258 | 1 | 45.78 |
| 213 | SLYD 104 | *S. lycopersicoides* | indel | - | 173 | 1 | 48.29 |
| 214 | SLYD 105 | *S. lycopersicoides* | indel | - | 251 | 1 | 52.49 |
| 215 | SLYD 95 | *S. lycopersicoides* | indel | - | 285 | 1 | 54.53 |
| 216 | SLYD 106 | *S. lycopersicoides* | indel | - | 196 | 1 | 57.89 |
| 217 | SLYD 107 | *S. lycopersicoides* | indel | - | 285 | 1 | 60.47 |
| 218 | SLYD 97 | *S. lycopersicoides* | indel | - | 198 | 1 | 64.15 |
| 219 | SLYD 110 | *S. lycopersicoides* | indel | - | 227 | 1 | 66.80 |
| 220 | SLYD 111 | *S. lycopersicoides* | indel | - | 269 | 1 | 69.25 |
| 221 | SLYD 98 | *S. lycopersicoides* | indel | - | 254 | 1 | 75.19 |
| 222 | SLYD 12 | *S. lycopersicoides* | indel | - | 352 | 1 | 79.10 |
| 223 | SLYD 99 | *S. lycopersicoides* | indel | - | 257 | 1 | 83.96 |
| 224 | SLYD 100 | *S. lycopersicoides* | indel | - | 172 | 1 | 86.88 |
| 225 | SLYD 101 | *S. lycopersicoides* | indel | - | 221 | 1 | 89.51 |
| 226 | SLYD 102 | *S. lycopersicoides* | indel | - | 298 | 1 | 91.10 |
| 227 | SLYD 13 | *S. lycopersicoides* | indel | - | 218 | 1 | 93.22 |
| 228 | SLYD 14 | *S. lycopersicoides* | indel | - | 317 | 2 | 0.04 |
| 229 | SLYD 113 | *S. lycopersicoides* | indel | - | 194 | 2 | 3.80 |
| 230 | SLYD 114 | *S. lycopersicoides* | indel | - | 141 | 2 | 6.94 |
| 231 | SLYD 15 | *S. lycopersicoides* | indel | - | 273 | 2 | 9.26 |
| 232 | SLYD 16 | *S. lycopersicoides* | indel | - | 374 | 2 | 15.60 |
| 233 | SLYD 17 | *S. lycopersicoides* | indel | - | 143 | 2 | 18.13 |
| 234 | SLYD 115 | *S. lycopersicoides* | indel | - | 216 | 2 | 18.84 |
| 235 | SLYD 18 | *S. lycopersicoides* | indel | - | 297 | 2 | 24.10 |
| 236 | SLYD 04 | *S. lycopersicoides* | indel | - | 339 | 2 | 27.25 |
| 237 | SLYD 116 | *S. lycopersicoides* | indel | - | 157 | 2 | 33.99 |
| 238 | SLYD 05 | *S. lycopersicoides* | indel | - | 347 | 2 | 36.06 |
| 239 | SLYD 117 | *S. lycopersicoides* | indel | - | 206 | 2 | 42.60 |
| 240 | SLYD 06 | *S. lycopersicoides* | indel | - | 339 | 2 | 45.01 |
| 241 | SLYD 118 | *S. lycopersicoides* | indel | - | 222 | 2 | 48.61 |
| 242 | SLYD 07 | *S. lycopersicoides* | indel | - | 350 | 2 | 54.05 |
| 243 | SLYD 08 | *S. lycopersicoides* | indel | - | 237 | 3 | 0.01 |
| 244 | SLYD 19 | *S. lycopersicoides* | indel | - | 390 | 3 | 3.02 |
| 245 | SLYD 20 | *S. lycopersicoides* | indel | - | 227 | 3 | 6.22 |
| 246 | SLYD 119 | *S. lycopersicoides* | indel | - | 261 | 3 | 12.84 |
| 247 | SLYD 21 | *S. lycopersicoides* | indel | - | 362 | 3 | 18.36 |
| 248 | SLYD 22 | *S. lycopersicoides* | indel | - | 279 | 3 | 24.02 |
| 249 | SLYD 120 | *S. lycopersicoides* | indel | - | 179 | 3 | 30.56 |
| 250 | SLYD 121 | *S. lycopersicoides* | indel | - | 241 | 3 | 36.17 |
| 251 | SLYD 23 | *S. lycopersicoides* | indel | - | 204 | 3 | 51.68 |
| 252 | SLYD 122 | *S. lycopersicoides* | indel | - | 218 | 3 | 54.01 |
| 253 | SLYD 123 | *S. lycopersicoides* | indel | - | 244 | 3 | 60.31 |
| 254 | SLYD 124 | *S. lycopersicoides* | indel | - | 150 | 3 | 66.11 |
| 255 | SLYD 125 | *S. lycopersicoides* | indel | - | 190 | 3 | 72.26 |
| 256 | SLYD 24 | *S. lycopersicoides* | indel | - | 309 | 4 | 0.03 |
| 257 | SLYD 126 | *S. lycopersicoides* | indel | - | 224 | 4 | 6.61 |
| 258 | SLYD 25 | *S. lycopersicoides* | indel | - | 377 | 4 | 12.30 |
| 259 | SLYD 127 | *S. lycopersicoides* | indel | - | 218 | 4 | 18.75 |
| 260 | SLYD 128 | *S. lycopersicoides* | indel | - | 230 | 4 | 24.25 |
| 261 | SLYD 26 | *S. lycopersicoides* | indel | - | 244 | 4 | 24.31 |
| 262 | SLYD 27 | *S. lycopersicoides* | indel | - | 204 | 4 | 30.44 |
| 263 | SLYD 28 | *S. lycopersicoides* | indel | - | 252 | 4 | 47.98 |
| 264 | SLYD 29 | *S. lycopersicoides* | indel | - | 238 | 5 | 0.13 |
| 265 | SLYD 129 | *S. lycopersicoides* | indel | - | 195 | 5 | 6.01 |
| 266 | SLYD 130 | *S. lycopersicoides* | indel | - | 257 | 5 | 12.57 |
| 267 | SLYD 30 | *S. lycopersicoides* | indel | - | 270 | 5 | 18.74 |
| 268 | SLYD 131 | *S. lycopersicoides* | indel | - | 207 | 5 | 21.23 |
| 269 | SLYD 31 | *S. lycopersicoides* | indel | - | 392 | 5 | 33.41 |
| 270 | SLYD 132 | *S. lycopersicoides* | indel | - | 265 | 5 | 36.95 |
| 271 | SLYD 133 | *S. lycopersicoides* | indel | - | 264 | 5 | 42.27 |
| 272 | SLYD 32 | *S. lycopersicoides* | indel | - | 208 | 5 | 45.13 |
| 273 | SLYD 134 | *S. lycopersicoides* | indel | - | 295 | 5 | 48.67 |
| 274 | SLYD 135 | *S. lycopersicoides* | indel | - | 142 | 5 | 51.04 |
| 275 | SLYD 136 | *S. lycopersicoides* | indel | - | 287 | 5 | 53.55 |
| 276 | SLYD 137 | *S. lycopersicoides* | indel | - | 178 | 5 | 57.01 |
| 277 | SLYD 33 | *S. lycopersicoides* | indel | - | 272 | 5 | 57.42 |
| 278 | SLYD 138 | *S. lycopersicoides* | indel | - | 180 | 5 | 63.02 |
| 279 | SLYD 34 | *S. lycopersicoides* | indel | - | 270 | 6 | 3.17 |
| 280 | SLYD 35 | *S. lycopersicoides* | indel | - | 323 | 6 | 6.75 |
| 281 | SLYD 139 | *S. lycopersicoides* | indel | - | 275 | 6 | 8.88 |
| 282 | SLYD 36 | *S. lycopersicoides* | indel | - | 267 | 6 | 15.51 |
| 283 | SLYD 140 | *S. lycopersicoides* | indel | - | 185 | 6 | 21.11 |
| 284 | SLYD 37 | *S. lycopersicoides* | indel | - | 362 | 6 | 24.86 |
| 285 | SLYD 38 | *S. lycopersicoides* | indel | - | 277 | 6 | 30.52 |
| 286 | SLYD 141 | *S. lycopersicoides* | indel | - | 269 | 6 | 36.00 |
| 287 | SLYD 142 | *S. lycopersicoides* | indel | - | 294 | 6 | 42.03 |
| 288 | SLYD 143 | *S. lycopersicoides* | indel | - | 179 | 6 | 48.00 |
| 289 | SLYD 39 | *S. lycopersicoides* | indel | - | 336 | 7 | 3.37 |
| 290 | SLYD 40 | *S. lycopersicoides* | indel | - | 254 | 7 | 9.13 |
| 291 | SLYD 144 | *S. lycopersicoides* | indel | - | 274 | 7 | 9.22 |
| 292 | SLYD 145 | *S. lycopersicoides* | indel | - | 293 | 7 | 12.47 |
| 293 | SLYD 41 | *S. lycopersicoides* | indel | - | 413 | 7 | 15.54 |
| 294 | SLYD 146 | *S. lycopersicoides* | indel | - | 241 | 7 | 21.86 |
| 295 | SLYD 42 | *S. lycopersicoides* | indel | - | 355 | 7 | 24.13 |
| 296 | SLYD 147 | *S. lycopersicoides* | indel | - | 142 | 7 | 30.13 |
| 297 | SLYD 148 | *S. lycopersicoides* | indel | - | 280 | 7 | 33.89 |
| 298 | SLYD 43 | *S. lycopersicoides* | indel | - | 306 | 7 | 36.28 |
| 299 | SLYD 149 | *S. lycopersicoides* | indel | - | 156 | 7 | 39.62 |
| 300 | SLYD 150 | *S. lycopersicoides* | indel | - | 135 | 7 | 42.66 |
| 301 | SLYD 44 | *S. lycopersicoides* | indel | - | 398 | 8 | 3.42 |
| 302 | SLYD 45 | *S. lycopersicoides* | indel | - | 469 | 8 | 9.17 |
| 303 | SLYD 46 | *S. lycopersicoides* | indel | - | 316 | 8 | 24.47 |
| 304 | SLYD 47 | *S. lycopersicoides* | indel | - | 229 | 8 | 33.47 |
| 305 | SLYD 48 | *S. lycopersicoides* | indel | - | 358 | 8 | 45.28 |
| 306 | SLYD 49 | *S. lycopersicoides* | indel | - | 298 | 9 | 3.24 |
| 307 | SLYD 50 | *S. lycopersicoides* | indel | - | 230 | 9 | 12.33 |
| 308 | SLYD 51 | *S. lycopersicoides* | indel | - | 370 | 9 | 18.63 |
| 309 | SLYD 52 | *S. lycopersicoides* | indel | - | 372 | 9 | 27.98 |
| 310 | SLYD 53 | *S. lycopersicoides* | indel | - | 293 | 9 | 33.70 |
| 311 | SLYD 54 | *S. lycopersicoides* | indel | - | 335 | 10 | 3.02 |
| 312 | SLYD 58 | *S. lycopersicoides* | indel | - | 209 | 10 | 8.46 |
| 313 | SLYD 55 | *S. lycopersicoides* | indel | - | 342 | 10 | 40.42 |
| 314 | SLYD 56 | *S. lycopersicoides* | indel | - | 265 | 10 | 46.07 |
| 315 | SLYD 57 | *S. lycopersicoides* | indel | - | 188 | 10 | 51.88 |
| 316 | SLYD 59 | *S. lycopersicoides* | indel | - | 275 | 11 | 0.09 |
| 317 | SLYD 60 | *S. lycopersicoides* | indel | - | 264 | 11 | 2.99 |
| 318 | SLYD 61 | *S. lycopersicoides* | indel | - | 229 | 11 | 5.53 |
| 319 | SLYD 62 | *S. lycopersicoides* | indel | - | 280 | 11 | 8.30 |
| 320 | SLYD 63 | *S. lycopersicoides* | indel | - | 261 | 11 | 11.78 |
| 321 | SLYD 64 | *S. lycopersicoides* | indel | - | 397 | 11 | 14.43 |
| 322 | SLYD 65 | *S. lycopersicoides* | indel | - | 311 | 11 | 17.17 |
| 323 | SLYD 66 | *S. lycopersicoides* | indel | - | 371 | 11 | 20.04 |
| 324 | SLYD 67 | *S. lycopersicoides* | indel | - | 209 | 11 | 23.16 |
| 325 | SLYD 68 | *S. lycopersicoides* | indel | - | 31 | 11 | 26.47 |
| 326 | SLYD 69 | *S. lycopersicoides* | indel | - | 328 | 11 | 28.27 |
| 327 | SLYD 70 | *S. lycopersicoides* | indel | - | 403 | 11 | 31.59 |
| 328 | SLYD 71 | *S. lycopersicoides* | indel | - | 245 | 11 | 34.19 |
| 329 | SLYD 72 | *S. lycopersicoides* | indel | - | 281 | 11 | 36.83 |
| 330 | SLYD 85 | *S. lycopersicoides* | indel | - | 120 | 11 | 38.15 |
| 331 | SLYD 73 | *S. lycopersicoides* | indel | - | 219 | 11 | 40.04 |
| 332 | SLYD 74 | *S. lycopersicoides* | indel | - | 83 | 11 | 42.27 |
| 333 | SLYD 75 | *S. lycopersicoides* | indel | - | 258 | 11 | 45.61 |
| 334 | SLYD 86 | *S. lycopersicoides* | indel | - | 334 | 11 | 47.53 |
| 335 | SLYD 76 | *S. lycopersicoides* | indel | - | 216 | 11 | 49.05 |
| 336 | SLYD 77 | *S. lycopersicoides* | indel | - | 210 | 11 | 51.22 |
| 337 | SLYD 78 | *S. lycopersicoides* | indel | - | 263 | 11 | 53.94 |
| 338 | SLYD 79 | *S. lycopersicoides* | indel | - | 229 | 11 | 56.56 |
| 339 | SLYD 87 | *S. lycopersicoides* | indel | - | 217 | 12 | 0.01 |
| 340 | SLYD 88 | *S. lycopersicoides* | indel | - | 187 | 12 | 3.27 |
| 341 | SLYD 89 | *S. lycopersicoides* | indel | - | 300 | 12 | 6.54 |
| 342 | SLYD 90 | *S. lycopersicoides* | indel | - | 210 | 12 | 9.35 |
| 343 | SLYD 91 | *S. lycopersicoides* | indel | - | 184 | 12 | 12.02 |
| 344 | SLYD 92 | *S. lycopersicoides* | indel | - | 245 | 12 | 15.39 |
| 345 | SLYD 93 | *S. lycopersicoides* | indel | - | 254 | 12 | 24.54 |

*^a^S. lycopersicoides*-specific SSRs

^b^*S. lycopersicoides*-specific indel markers

^c^simple sequence repeats

^d^insertion/deletion

^e^Chr = chromosome number

^f^Map position is based on tomato chromosomes as a reference

^g^No specific blast hit in tomato reference but amplified multiple locus in *S. lycopersicoides*
