# Supplementary material for: Development of a PCR-based, genetic marker resource for the tomato-like nightshade relative, Solanum lycopersicoides using whole genome sequence analysis
Source: PLoS One. 2020 Nov 23;15(11):e0242882. doi: 10.1371/journal.pone.0242882 (PMC7682897; doi:10.1371/journal.pone.0242882)
Supplement: S2 Table — (DOCX) [file pone.0242882.s002.docx]

**S2 Table.** **Forward and reverse primer sequences of *S. lycopersicoides*-specific markers.**

| No. | Primer name | Source Species | Forward primer | Reverse primer | T_A_^c^ (ᵒC) |
| --- | --- | --- | --- | --- | --- |
| 1 | SLM^a^ 01 | *S. lycopersicoides* | tttccgtaccgccctcaaaa | attcaccacttcaccacccc | 55 |
| 2 | SLM 02 | *S. lycopersicoides* | tggtttgtgtggaagacgaa | caacccaaacgaccaaagct | 55 |
| 3 | SLM 03 | *S. lycopersicoides* | tggaccgtaattgcgaggaa | ttaaccctcctgcgatctgc | 55 |
| 4 | SLM 04 | *S. lycopersicoides* | taatcacttgtggtgccgct | aagcacttcgccacctacaa | 55 |
| 5 | SLM 05 | *S. lycopersicoides* | ctaatgtagtgtggaggaac | tacacacttgttatccactc | 53 |
| 6 | SLM 06 | *S. lycopersicoides* | aatgccctcacactccttcg | gggattatttggattccaag | 53 |
| 7 | SLM 07 | *S. lycopersicoides* | attgagttcctcttcctcttcc | gttgggcaacaaaccaagag | 53 |
| 8 | SLM 08 | *S. lycopersicoides* | tgccccataggaaacctagc | gtcgccggttacgtctagg | 55 |
| 9 | SLM 09 | *S. lycopersicoides* | ggagcatgctagttggggtt | tcaaatctgggtagacgcct | 55 |
| 10 | SLM 10 | *S. lycopersicoides* | acgcaatcctgacagatactga | acgtaggcggcactaaagtt | 55 |
| 11 | SLM 11 | *S. lycopersicoides* | ccctagatgtaaccggcgtc | tgcttcctattgcgagaagtct | 55 |
| 12 | SLM 12 | *S. lycopersicoides* | gcccaactcgcaatcctcta | aggttaacatagagctctcgca | 55 |
| 13 | SLM 13 | *S. lycopersicoides* | ctccgtttcaagttaagttaggtca | tgcaagtttaacgtgaggaga | 55 |
| 14 | SLM 14 | *S. lycopersicoides* | cgcaacaattcaggcccaat | acgttcttcaactccttccgt | 55 |
| 15 | SLM 15 | *S. lycopersicoides* | agcatgaggactttacttgca | acggtgccaaacttccacta | 55 |
| 16 | SLM 16 | *S. lycopersicoides* | agatttgtgggctgaaccgt | tcccaccatttgcagtgaca | 55 |
| 17 | SLM 17 | *S. lycopersicoides* | acggtgattttcctgctaaga | attctgactcaccggccaag | 55 |
| 18 | SLM 18 | *S. lycopersicoides* | agtgacactcataacagatcgga | ccgcgcaacaagtacaaaca | 55 |
| 19 | SLM 19 | *S. lycopersicoides* | tgccccacctacaaacacaa | aacccttaaaccctcctgcg | 55 |
| 20 | SLM 20 | *S. lycopersicoides* | gggctctcctcctatataaagagc | tgggattgagtgagtcccct | 55 |
| 21 | SLM 21 | *S. lycopersicoides* | ttccgtgccacagtgttgta | cccctgccccttgttactac | 55 |
| 22 | SLM 22 | *S. lycopersicoides* | gggaagcaagatgttgggga | ggagaaaggaaggaggaagttgt | 55 |
| 23 | SLM 23 | *S. lycopersicoides* | tccgctcttcaggtaatgca | gctaggcagccattaaggact | 55 |
| 24 | SLM 24 | *S. lycopersicoides* | cgcacacactgctacagaga | cagctgcagcaatggcattt | 55 |
| 25 | SLM 25 | *S. lycopersicoides* | aaaagtgtcaatgagcccgc | tttgccgtcatttgcagacg | 55 |
| 26 | SLM 26 | *S. lycopersicoides* | ccattcacacttgctaaaccca | tgacaccgcagtttaaacca | 55 |
| 27 | SLM 27 | *S. lycopersicoides* | tgggtagatggtggggagtt | acgttaatgacacgtaaccaac | 55 |
| 28 | SLM 28 | *S. lycopersicoides* | atgtgtgagtaggaccaaatgt | caatcagaaacaactcaaacaccc | 55 |
| 29 | SLM 29 | *S. lycopersicoides* | tgatggtttgtgatcatctctcct | aaaacagcctcactcccacc | 55 |
| 30 | SLM 30 | *S. lycopersicoides* | tgtcgccatcataggttactagc | acctcaccctttggtcatcc | 55 |
| 31 | SLM 31 | *S. lycopersicoides* | aagcctccttcttcatgggc | gggttaaccgtggaccagtt | 55 |
| 32 | SLM 32 | *S. lycopersicoides* | acccacctacactctaagtctga | cgaccgggatgaaccatctt | 55 |
| 33 | SLM 33 | *S. lycopersicoides* | ggggcatgagttcaggtgaa | aattgcaccccatcacgtct | 55 |
| 34 | SLM 34 | *S. lycopersicoides* | tgatcgctctcctgttgtcc | ccgaaagtataaagaggcacttgg | 55 |
| 35 | SLM 35 | *S. lycopersicoides* | tggggtaagtgacattccct | cactcaacatgccaaagctca | 55 |
| 36 | SLM 36 | *S. lycopersicoides* | agaggagcctaagatttgaaggt | ccagagtggtttaattgctcct | 55 |
| 37 | SLM 37 | *S. lycopersicoides* | gagttcccactcgtgaccag | aggaggttggtctgccctaa | 55 |
| 38 | SLM 38 | *S. lycopersicoides* | tgttttagtgtgtgtgtgtgtg | gctcgtgaaatcatactcggc | 55 |
| 39 | SLM 39 | *S. lycopersicoides* | tggcatggtatgtttccaca | gtggaattgtgtcaatgactgca | 55 |
| 40 | SLM 40 | *S. lycopersicoides* | tgacacccatcacaaagaagga | gcttcagcatcagtccagga | 55 |
| 41 | SLM 41 | *S. lycopersicoides* | catagtggtggctgtcggtt | cgatgcaagccaacaagcat | 55 |
| 42 | SLM 42 | *S. lycopersicoides* | gcttctagcactttctagccca | gtgtcaaaggggcatcggta | 55 |
| 43 | SLM 43 | *S. lycopersicoides* | tcaagatggggagttcatgct | agtgggttactagtttaccgagt | 55 |
| 44 | SLM 44 | *S. lycopersicoides* | acataggtcctggtggtggt | acccgacatttatcgtgcct | 55 |
| 45 | SLM 45 | *S. lycopersicoides* | aagacgtcatttgcctgttgg | tattgggccagtgctagcac | 55 |
| 46 | SLM 46 | *S. lycopersicoides* | ggttattcaagtggctaccagc | attttccgacgagacacccc | 55 |
| 47 | SLM 47 | *S. lycopersicoides* | tgcaagaagtatatcctgcctca | tgtcgtcatgaagtaagtggca | 55 |
| 48 | SLM 48 | *S. lycopersicoides* | tgggagagagaagaggtgtgt | tcttttctctcgggaccaattt | 55 |
| 48 | SLM 49 | *S. lycopersicoides* | gttggtggagctgaaagaagc | tcagctcaggatgcttctca | 55 |
| 50 | SLM 50 | *S. lycopersicoides* | acctgtctgaacgtgacatcc | tgcaggagcttcatttccca | 55 |
| 51 | SLM 51 | *S. lycopersicoides* | agattgaggggagtggtcga | gaacctgggtgaagctcctc | 55 |
| 52 | SLM 52 | *S. lycopersicoides* | gcattctgcactaccctatcac | agtgtgtgaccaaacgagca | 55 |
| 53 | SLM 53 | *S. lycopersicoides* | agcaaatgaagtactctccctagt | tcatttgtccgatcctttctca | 55 |
| 54 | SLM 54 | *S. lycopersicoides* | agaaaagaagaagcggccaa | acataacaaatctcgctcgcc | 55 |
| 55 | SLM 55 | *S. lycopersicoides* | aggctctctactcttcggcc | aggtcttcaccaatgctgca | 55 |
| 56 | SLM 56 | *S. lycopersicoides* | tctctcctttcacgttcccc | acccagagtgcaaaatttcga | 55 |
| 57 | SLM 57 | *S. lycopersicoides* | tctttctctagtcccaatgcaaga | aggatcaacagctaaaacaggga | 55 |
| 58 | SLM 58 | *S. lycopersicoides* | tggattggttgttggcttagt | tgcacaaccaccatatgtgt | 55 |
| 59 | SLM 59 | *S. lycopersicoides* | aaggaccacgttgcacttca | caagtaaccgtatctagcttgatga | 55 |
| 60 | SLM 60 | *S. lycopersicoides* | tggtgaagatttaggctttgct | cggtaattgacacttgtcttcaca | 55 |
| 61 | SLM 61 | *S. lycopersicoides* | tttgttggcaattcagggga | tgcaccaacgtggaaatgtt | 55 |
| 52 | SLM 62 | *S. lycopersicoides* | tctgtaaactatgtcgccagca | tcctagccgaacactcctca | 55 |
| 63 | SLM 63 | *S. lycopersicoides* | aacaacgagatccaccagcg | agatccatggcctttgggtg | 55 |
| 64 | SLM 64 | *S. lycopersicoides* | tgaactctcgctaacaacact | agctcttggcaccttcagtt | 55 |
| 65 | SLM 65 | *S. lycopersicoides* | tccggaccttagtaccacga | acaagaaacgtgtgaagcgc | 55 |
| 66 | SLM 66 | *S. lycopersicoides* | accctcagctcttgcagaaa | tggttgatagtgggagtcgt | 55 |
| 67 | SLM 67 | *S. lycopersicoides* | gaccaacttcgttcattggca | acttgagcaagaccaagggt | 55 |
| 68 | SLM 68 | *S. lycopersicoides* | gccatcggataagccctcaa | tgcaatgaacgacaaagcgg | 55 |
| 69 | SLM 69 | *S. lycopersicoides* | ggcctcttttctcccctgac | tcctccgtgatctgggtgat | 55 |
| 70 | SLM 70 | *S. lycopersicoides* | tgtatgatgatggttcttgtagtcc | gtgtgtttaaactaagcccacca | 55 |
| 71 | SLM 71 | *S. lycopersicoides* | ttgggtcagcaagaacgaaa | cgatgcaatattcatgcggga | 55 |
| 72 | SLM 72 | *S. lycopersicoides* | gctggagagcatggaaggtt | cctactcattcgggttcgagt | 55 |
| 73 | SLM 73 | *S. lycopersicoides* | cccacactgcacccacaaaa | gggaggccagatgcagaata | 55 |
| 74 | SLM 74 | *S. lycopersicoides* | tacaccttcgtcgagatccc | cccaacctcattccttccgt | 55 |
| 75 | SLM 75 | *S. lycopersicoides* | gtgtttgtttaacgcgagaaagag | cgcgttaggatagagtgggg | 55 |
| 76 | SLM 76 | *S. lycopersicoides* | ccacagctgaggcatggtaa | acggcttcttgcttcaacca | 55 |
| 77 | SLM 77 | *S. lycopersicoides* | tgtctccctttgaccatgtaca | tgagaagttttcttgtctgctactg | 55 |
| 78 | SLM 78 | *S. lycopersicoides* | ggaggagctcacatgaaccc | gtgtctgtgtgtgggagagg | 55 |
| 79 | SLM 79 | *S. lycopersicoides* | tccgcattaataggcacaca | tattcactgcggcacaccat | 55 |
| 80 | SLM 80 | *S. lycopersicoides* | tgaccatcatacatgtctcaaagtg | cttacttaatgagcattacacctcc | 55 |
| 81 | SLM 81 | *S. lycopersicoides* | cccaaaccctaaccctaaccc | ggtagtttgggcttggggtt | 55 |
| 82 | SLM 82 | *S. lycopersicoides* | gagaaccaaccatgtagggca | tcagccacctctacccgaat | 55 |
| 83 | SLM 83 | *S. lycopersicoides* | tccacaactgcaagaccctc | cgtggccttttcttgttcgt | 55 |
| 84 | SLM 84 | *S. lycopersicoides* | tgtaggtgagggtatgggact | tgcatctaaagtgagacctcatca | 55 |
| 85 | SLM 85 | *S. lycopersicoides* | ttgccacatcagcggactta | atgatgtggcacttacgtgg | 55 |
| 86 | SLM 86 | *S. lycopersicoides* | attgtagggtccgggaaagc | ggggctacctaacatttgttgt | 55 |
| 87 | SLM 87 | *S. lycopersicoides* | gccagtaaccctatcacgca | accctagccatgaattgcaca | 55 |
| 88 | SLM 88 | *S. lycopersicoides* | ttgccctgatcctccaacac | tccccaataattagccccgc | 55 |
| 89 | SLM 89 | *S. lycopersicoides* | ggaggtcaatggaaaacctgg | agtatgcactacggacacaaa | 55 |
| 90 | SLM 90 | *S. lycopersicoides* | acaaactgtgtgctacgtgt | ccacagtctgatgctccatga | 55 |
| 91 | SLM 91 | *S. lycopersicoides* | acctcacgacgccttcaatt | cctcacaaggtctcgtttca | 55 |
| 92 | SLM 92 | *S. lycopersicoides* | aagtgaggggtcttttgggt | tgaaggacctattggggaaagg | 53 |
| 93 | SLM 93 | *S. lycopersicoides* | agacgttcttggacgttttagt | aagagaaccctaggcccacc | 55 |
| 94 | SLM 94 | *S. lycopersicoides* | ggaacggagtcattgctaca | gctccattccgacgaggttt | 53 |
| 95 | SLM 95 | *S. lycopersicoides* | tggagttggagccacttgaa | gaagactgtacaaaccacaaagaag | 53 |
| 96 | SLM 96 | *S. lycopersicoides* | gtgctgtctgtggagctgtt | gcacttgagccgagagtctt | 55 |
| 97 | SLM 97 | *S. lycopersicoides* | aagcaaagcaaggaggagga | aggaagactcgggagaataca | 55 |
| 98 | SLM 98 | *S. lycopersicoides* | gctcaagggctagcatccaa | aattgaggggaagccaccag | 55 |
| 99 | SLM 99 | *S. lycopersicoides* | gatgaaaggactgcatcgcc | ggtgtttctattcgtacttcccg | 55 |
| 100 | SLM 100 | *S. lycopersicoides* | actcggtattggtgtatcccc | ttgttgattgtgccgtttgc | 55 |
| 101 | SLM 101 | *S. lycopersicoides* | tgtgatgctgacacgtaatcg | ccccttaatgttgggctcgt | 55 |
| 102 | SLM 102 | *S. lycopersicoides* | cttcagtccagctccaaccc | actccactgaactcccaatcag | 55 |
| 103 | SLM 103 | *S. lycopersicoides* | gctatggattgtcccgccaa | tccatgaggaaaacgacaaagt | 55 |
| 104 | SLM 104 | *S. lycopersicoides* | tggacaagttctgctcttctcc | gtatgaaggtagctccgccc | 55 |
| 105 | SLM 105 | *S. lycopersicoides* | ggacacacacaaacacgtgg | acaacatgaagcccaacctt | 53 |
| 106 | SLM 106 | *S. lycopersicoides* | atcaagctaccacgcaccat | ccggaccctttgatttcgct | 55 |
| 107 | SLM 107 | *S. lycopersicoides* | ctgtcggttcagcgatgaga | agcacatagggatcgatcaga | 55 |
| 108 | SLM 108 | *S. lycopersicoides* | ggaagtgtgctttgtcgagg | ggcaaaccatacgatccaaacc | 55 |
| 109 | SLM 109 | *S. lycopersicoides* | gcaatacaagccaggggtct | gtgcctcccttttgattggc | 53 |
| 110 | SLM 110 | *S. lycopersicoides* | caccacactatgtattaaaggtgac | ggatgagcttgtacgtgcct | 55 |
| 111 | SLM 111 | *S. lycopersicoides* | acgatatcccatcaacagctga | ggcaactagggaggctacag | 55 |
| 112 | SLM 112 | *S. lycopersicoides* | cccatgacaacccactaccc | tgggttgatataactgttgggt | 55 |
| 113 | SLM 113 | *S. lycopersicoides* | gacagagatgaaggtggagca | gccacaacgatgaatcaacca | 55 |
| 114 | SLM 114 | *S. lycopersicoides* | ggggaagggagtgagatgaa | ccttgttgtgactctttgcatgt | 55 |
| 115 | SLM 115 | *S. lycopersicoides* | ggaagtatggagggtggcac | gagaggtgagagcaattgggt | 55 |
| 116 | SLM 116 | *S. lycopersicoides* | tctgtgccctcaaccttcac | cggcacagctcgatatgact | 55 |
| 117 | SLM 117 | *S. lycopersicoides* | tgtagcgaaggacgatcattc | ggtcagcagggcagaatctt | 53 |
| 118 | SLM 118 | *S. lycopersicoides* | tccatggatgtctcttttcccc | ccctcacatcgaaaggaggg | 55 |
| 119 | SLM 119 | *S. lycopersicoides* | tcgagaacattcggaactgaaga | tccaaacattaacattgccgca | 55 |
| 120 | SLM 120 | *S. lycopersicoides* | cttggtgcgaattccgtgtg | attttgcggtgcttgccatc | 55 |
| 121 | SLM 121 | *S. lycopersicoides* | aagctcaggttgtggctgtt | gcctcaattgccccaggaat | 55 |
| 122 | SLM 122 | *S. lycopersicoides* | tggcaaccaagacccacaat | ccatcgagccaaaaggggaa | 55 |
| 123 | SLM 123 | *S. lycopersicoides* | tgtattcaacaagcctcgggg | aagggaaacaaaatgagtgaccac | 53 |
| 124 | SLM 124 | *S. lycopersicoides* | gggtacaatgctagcctacca | ccacatatgcgggcctagag | 55 |
| 125 | SLM 125 | *S. lycopersicoides* | gtgggactctatgcaggaca | gctcttgtacaaggttcccct | 55 |
| 126 | SLM 126 | *S. lycopersicoides* | ccaatcatatgcttcaaaaggtcgt | tggtgtttgactttcagctct | 53 |
| 127 | SLM 127 | *S. lycopersicoides* | gcatgaatcctgcatgtaagca | aggaaggaagactctctctctct | 53 |
| 128 | SLM 128 | *S. lycopersicoides* | tccatgatggtggtcaaagct | acgcttgggacagaagagtt | 53 |
| 129 | SLM 129 | *S. lycopersicoides* | ctgctcctccctaatgcaca | gtgcaccttactcggacgaa | 53 |
| 130 | SLM 130 | *S. lycopersicoides* | aggtaagccacaccaccaac | gctcccctgagaccaacttc | 55 |
| 131 | SLM 131 | *S. lycopersicoides* | ccaatgagaatcaccctattgagg | gctaaagccctgatggagct | 53 |
| 132 | SLM 132 | *S. lycopersicoides* | gcaggggtaaaatgtcttgtcc | acagacgtgggaatatggaac | 53 |
| 133 | SLM 133 | *S. lycopersicoides* | agtttgtctagtggctacctgt | tgagaggccgaaagaaagtaca | 55 |
| 134 | SLM 134 | *S. lycopersicoides* | tggtcacatttcaaggtccaa | tcccctctttctcacgaggt | 53 |
| 135 | SLM 135 | *S. lycopersicoides* | tggactgtgtatatgtggtttgtg | gcatcgtgccattgaaccaa | 53 |
| 136 | SLM 136 | *S. lycopersicoides* | tccatggttgtctgtcatgct | acaactcctctaccttctcaaga | 55 |
| 137 | SLM 137 | *S. lycopersicoides* | gtgggaccacagtaggttgg | ctttggtgcaacccactgtg | 53 |
| 138 | SLM 138 | *S. lycopersicoides* | ctcttggtcttgtctgccgt | tgcaggtcaaatgacatcagt | 55 |
| 139 | SLM 139 | *S. lycopersicoides* | cttgccatgtgaacgagcag | acctgaatacatgtgtgacga | 53 |
| 140 | SLM 140 | *S. lycopersicoides* | ggcgatggcttccttgaaga | tctttagcagtgtgaattactaccc | 53 |
| 141 | SLM 141 | *S. lycopersicoides* | acaccttcaaagtgctaccca | aggggcactatctcacacaa | 55 |
| 142 | SLM 142 | *S. lycopersicoides* | ggaacttctgcattgtcccg | cgaattcaaaggtacaatctgcaga | 55 |
| 143 | SLM 143 | *S. lycopersicoides* | tgacatgtgcatgcaagtttga | atcgcctctagcctagggtt | 53 |
| 144 | SLM 144 | *S. lycopersicoides* | ggcaaacataggctcaaaagtca | tgtgaatcacgccatccaat | 53 |
| 145 | SLM 145 | *S. lycopersicoides* | tcaatgtgaaaggtggctgc | aaggtgtctcgttcgccaaa | 53 |
| 146 | SLM 146 | *S. lycopersicoides* | cgaggccagtccgcttaaaa | acggcctacacaaaccagag | 55 |
| 147 | SLM 147 | *S. lycopersicoides* | ggacatggtcaggctgagag | tgacacttcacctcgtctgt | 53 |
| 148 | SLM 148 | *S. lycopersicoides* | tcatcctttggtgggacctc | accacttcccttgtctgaga | 55 |
| 149 | SLM 149 | *S. lycopersicoides* | tggcctacatcatgtcaagca | caccgagggtcgccttttaa | 53 |
| 150 | SLM 150 | *S. lycopersicoides* | gctgcttgatgtacataagcaga | agaactttaaggttgctggct | 55 |
| 151 | SLM 151 | *S. lycopersicoides* | tcacacccatcatgttcatagca | acctgtgaatgtccctcttctc | 55 |
| 152 | SLM 152 | *S. lycopersicoides* | cacactttcatgtggtactcca | acatgcgatactgacttttcgt | 53 |
| 153 | SLM 153 | *S. lycopersicoides* | aaagatggcaagagagggcg | agccccatcagtctaggtgt | 55 |
| 154 | SLM 154 | *S. lycopersicoides* | aggcaccaatgactcacctt | acctattggatacgcgactgt | 55 |
| 155 | SLM 155 | *S. lycopersicoides* | cgaaatactcagaggacatgcc | tctccttgatcctggctcca | 53 |
| 156 | SLM 156 | *S. lycopersicoides* | tgatggagaagatgatcgacca | tctttggctctgtgttcccc | 55 |
| 157 | SLM 157 | *S. lycopersicoides* | agttcaaggactagcatccaaga | gccaccagcaagttgtacca | 55 |
| 158 | SLM 158 | *S. lycopersicoides* | aattctgttgctgctgctgc | ccttttagtttcttgattttcgcgc | 55 |
| 159 | SLM 159 | *S. lycopersicoides* | ccagggaatggggttgacaa | cgtctgatctcgacctgagc | 55 |
| 160 | SLM 160 | *S. lycopersicoides* | gccacctgatcccaatgagt | cgatcagctggtccatacgg | 55 |
| 161 | SLM 161 | *S. lycopersicoides* | cgcgcaagtggaagaaacaa | tcacgaggcaacattctaacca | 53 |
| 162 | SLM 162 | *S. lycopersicoides* | tgacaagtgactaaacaccaaacg | cgaactagatatccccaggatgt | 55 |
| 163 | SLM 163 | *S. lycopersicoides* | atgatgaaggtgcaagttgtg | tgactcaccttattgtgcatactca | 53 |
| 164 | SLM 164 | *S. lycopersicoides* | tgaggactccatggatgaaagc | tggcaatttggtctaggggtc | 55 |
| 165 | SLM 165 | *S. lycopersicoides* | tgattttgttgcacttcctcct | tggttggaagggtgtttcgt | 55 |
| 166 | SLM 166 | *S. lycopersicoides* | aggtaaattttgcagcggcg | cagaatccagctcgcattgc | 55 |
| 167 | SLM 167 | *S. lycopersicoides* | accttgactctttgggttgaa | atagttcaccactgcgcgaa | 55 |
| 168 | SLM 168 | *S. lycopersicoides* | cattggcaatgattaacagggt | tccaatcacttacaacaaccca | 55 |
| 169 | SLM 169 | *S. lycopersicoides* | gtgatttccatgacgacctcct | ttttgacctccctcttgcca | 55 |
| 170 | SLM 170 | *S. lycopersicoides* | ttggaggaacgttttgcctt | tgacttctgaaccccttctgg | 53 |
| 171 | SLM 171 | *S. lycopersicoides* | acctctctctggtggggaaa | tgaccctggctcattggatc | 55 |
| 172 | SLM 172 | *S. lycopersicoides* | gcagatattcttcacccattttgac | aactaaggaagtgagcattagcttc | 53 |
| 173 | SLM 173 | *S. lycopersicoides* | ctcgggagtgcttcttggat | accaaagcttccgttcctca | 55 |
| 174 | SLM 174 | *S. lycopersicoides* | aactctggctcatccacgtg | gacagagacagggacaggga | 55 |
| 175 | SLM 175 | *S. lycopersicoides* | tggcttagtattgtgtacatggct | cttcacatagttcacctatacaagc | 55 |
| 176 | SLM 176 | *S. lycopersicoides* | agcgcttattcctctccaca | tgtgaaagaaagcatgccca | 53 |
| 177 | SLM 177 | *S. lycopersicoides* | cagtgttgactgggagcttt | catgttgtagcgttgcagcc | 55 |
| 178 | SLM 178 | *S. lycopersicoides* | tctgacgacccaacaacagc | aggatgcaaagcagtcctca | 55 |
| 179 | SLM 179 | *S. lycopersicoides* | agtgtacttgtcactaccatcca | gttcagtcacttccttattggca | 55 |
| 180 | SLM 180 | *S. lycopersicoides* | gttgattctttctgttccatcacac | ggctgaaccagatgcctcaa | 55 |
| 181 | SLM 181 | *S. lycopersicoides* | ctcatgggagatggacagcc | ggattgctggctttgctgtg | 55 |
| 182 | SLM 182 | *S. lycopersicoides* | accaactcactactcactactca | acaacaacctgagagccaca | 55 |
| 183 | SLM 183 | *S. lycopersicoides* | aaaagcctttgttccaccgc | tttcttcttcagctgcgggt | 53 |
| 184 | SLM 184 | *S. lycopersicoides* | gacatccacgtcctcagcaa | ttcggtgcagttcgttggat | 53 |
| 185 | SLM 185 | *S. lycopersicoides* | ccttgtgcagccaaacatcc | tggtggttacaacaacgaacg | 53 |
| 186 | SLM 186 | *S. lycopersicoides* | gcactcattgccgtgttgtg | tgcataaatggcacgagcag | 55 |
| 187 | SLM 187 | *S. lycopersicoides* | cgtttccccttgttttgcgt | gcaggtatcatgttggctgc | 55 |
| 188 | SLM 188 | *S. lycopersicoides* | gtaaagttacccgcgctcct | aatcttggtttgtggtggcg | 55 |
| 189 | SLM 189 | *S. lycopersicoides* | ccaaagaaatgtgacttttcctcag | tgctaagctttggactcactca | 55 |
| 190 | SLM 190 | *S. lycopersicoides* | gcctgaggctcacactttca | tgctgatgattgcctggaca | 55 |
| 191 | SLM 191 | *S. lycopersicoides* | tcagaagcgcctcaatttgc | acaagtcttcaaactctctctctct | 55 |
| 192 | SLM 192 | *S. lycopersicoides* | aggagcatgtgaagaagccg | tccttttctctttggctttatggc | 53 |
| 193 | SLM 193 | *S. lycopersicoides* | tggccagcaagtaaaatgcc | gttctttccatatatgcatgtcacg | 53 |
| 194 | SLM 194 | *S. lycopersicoides* | acgtatagaaagaagtgagacgtca | acacgtaaatctgcctcttcaca | 55 |
| 195 | SLM 195 | *S. lycopersicoides* | agggaagagattgaagagttagaga | tccatattttaatcccacgtgtcac | 55 |
| 196 | SLM 196 | *S. lycopersicoides* | ctgccaaaccctcaacaagc | aaggctcaccctcactctct | 55 |
| 197 | SLYD^b^ 01 | *S. lycopersicoides* | aggatcaatgtttgcccctg | gtgccaacaaacctgtgaag | 55 |
| 198 | SLYD 02 | *S. lycopersicoides* | ctcgttagtgcacccatttc | tgtttcccatagaacctcgg | 55 |
| 199 | SLYD 03 | *S. lycopersicoides* | tcacacatgagaggtaaccc | tgaatgtccagttctgccac | 55 |
| 200 | SLYD 04 | *S. lycopersicoides* | cgcttctccagctaaaatgc | tcgagaatgctggtatcagc | 55 |
| 201 | SLYD 05 | *S. lycopersicoides* | aacaacaaccctggctttgc | gagcagatggacagaacatc | 55 |
| 202 | SLYD 06 | *S. lycopersicoides* | attcggcaaatgcagctagc | tacacaactccggagttctc | 55 |
| 203 | SLYD 07 | *S. lycopersicoides* | gcactgaagactcttcttcc | cctacggatacagaagaacc | 55 |
| 204 | SLYD 08 | *S. lycopersicoides* | tcgaagaccaacctcctttc | tcaaactgccaacacctgtg | 55 |
| 205 | SLYD 09 | *S. lycopersicoides* | cgactaccttagaccaatgg | ccatccactacacctcaatc | 55 |
| 206 | SLYD 10 | *S. lycopersicoides* | tagggctctgttagaatgcg | tggaattgactgctggtgag | 55 |
| 207 | SLYD 11 | *S. lycopersicoides* | ggggaaggtaagatgtacag | tggcttgattgggaaggttg | 55 |
| 208 | SLYD 12 | *S. lycopersicoides* | ggggttcttgtttccatctc | cagtcgtcagacaactgtag | 55 |
| 209 | SLYD 13 | *S. lycopersicoides* | ttcagagacaccagcatcag | tactgtttcggtttggggac | 55 |
| 210 | SLYD 14 | *S. lycopersicoides* | tttggtcagtttaggcaggg | tctttacacgggagaagagg | 55 |
| 211 | SLYD 15 | *S. lycopersicoides* | cagactttgcctaggaagag | ggcttcaacggaagatcaag | 55 |
| 212 | SLYD 16 | *S. lycopersicoides* | aagatggatcccctcattgg | ggtagataggttggtgaagc | 55 |
| 213 | SLYD 17 | *S. lycopersicoides* | agtatgacggtgggatagag | gtcttgcacgaaccatactc | 55 |
| 214 | SLYD 18 | *S. lycopersicoides* | gtccacacatccatctcaac | cagtgcgacagatgtactag | 55 |
| 215 | SLYD 19 | *S. lycopersicoides* | tgagagttgcctatccagag | aacaatgccctctctgcatc | 55 |
| 216 | SLYD 20 | *S. lycopersicoides* | aggtggagagcttgtgaatg | ggtggagacatgattgtacg | 55 |
| 217 | SLYD 21 | *S. lycopersicoides* | tccattgtggtggctacaac | tcactaatccaccagtcagc | 55 |
| 218 | SLYD 22 | *S. lycopersicoides* | aacttcagaagccaagctgg | gagttgttgcatgcctagtg | 55 |
| 219 | SLYD 23 | *S. lycopersicoides* | tgccatatgggggtagaaac | tcatagggctgtgcttcatc | 55 |
| 220 | SLYD 24 | *S. lycopersicoides* | gtcccttacacatcgactag | gtgatcccactttcctgaac | 55 |
| 221 | SLYD 25 | *S. lycopersicoides* | cctggtttgtgtcaattggc | ttacccttggcctaacgatc | 55 |
| 222 | SLYD 26 | *S. lycopersicoides* | tattgcgcactcaaagtcgg | ctcctttggaagaaacaccg | 55 |
| 223 | SLYD 27 | *S. lycopersicoides* | ccttcaagagaagcaaggag | ccttggtaaattctgggagg | 55 |
| 224 | SLYD 28 | *S. lycopersicoides* | tgcgatgtctggatttgtgc | cctttggaaggaggtcattc | 55 |
| 225 | SLYD 29 | *S. lycopersicoides* | agacatccctgcaagcaatg | aaggcgaaatacccacttcc | 55 |
| 226 | SLYD 30 | *S. lycopersicoides* | gatgactgggtatctttcgc | ccatctgttggttgaggaag | 55 |
| 227 | SLYD 31 | *S. lycopersicoides* | ttggttgtcggtttcttccc | agagtgaggttgagcactag | 55 |
| 228 | SLYD 32 | *S. lycopersicoides* | agtcctcatgttgtgttccc | cctttgcgtatttaccctgg | 55 |
| 229 | SLYD 33 | *S. lycopersicoides* | ggtaatcctcatcagagctg | ccattttcgatgtcggagtc | 55 |
| 230 | SLYD 34 | *S. lycopersicoides* | ggcacaagttcacactactg | acttatgcctatgccttggg | 53 |
| 231 | SLYD 35 | *S. lycopersicoides* | tcatactccactgctagctc | acagtcttctcaggtcctag | 55 |
| 232 | SLYD 36 | *S. lycopersicoides* | aagacagaccaatgggagac | ttccacacactcagcttgag | 55 |
| 233 | SLYD 37 | *S. lycopersicoides* | agagtcacataccctttccc | atggcaaggggaggtatttg | 53 |
| 234 | SLYD 38 | *S. lycopersicoides* | tcccttgtctgtggttaagc | tcctcagcattaagggctac | 55 |
| 235 | SLYD 39 | *S. lycopersicoides* | gtctatcaacaagagcccag | tcccccaattcattgaccac | 55 |
| 236 | SLYD 40 | *S. lycopersicoides* | ttctcggggttctatacgtc | agtgtagggttcgagatgac | 55 |
| 237 | SLYD 41 | *S. lycopersicoides* | cgaacccaagcttagatcag | ccgtagatggaacctacaag | 55 |
| 238 | SLYD 42 | *S. lycopersicoides* | cattgtcggacatggaatgg | gagcaagagtgcttgtgtac | 55 |
| 239 | SLYD 43 | *S. lycopersicoides* | ggagggatatggcctttatg | caacacccgataggtaagtc | 55 |
| 240 | SLYD 44 | *S. lycopersicoides* | ggggttgtgggagaaataag | aacgaacaagacgagccaag | 55 |
| 241 | SLYD 45 | *S. lycopersicoides* | ccaattgggatgtatgagcc | ctctctgaagtgtagtagcc | 55 |
| 242 | SLYD 46 | *S. lycopersicoides* | atgacctcctccttcatgtc | agctgtttgcacactgaagg | 53 |
| 243 | SLYD 47 | *S. lycopersicoides* | gagctttgaggtcaaacgtc | agatcacccaatcttggcag | 55 |
| 244 | SLYD 48 | *S. lycopersicoides* | tcaacagcagtggaagactc | cttcgaggctcaacattcag | 55 |
| 245 | SLYD 49 | *S. lycopersicoides* | cggtactgatcagacatgtc | tattccagcagctgagaagg | 55 |
| 246 | SLYD 50 | *S. lycopersicoides* | tttcagtgccacaattgggc | aacaccacttgaagcttccc | 55 |
| 247 | SLYD 51 | *S. lycopersicoides* | tcctgtgtgcttgaaggatc | ccagcagctgaattcaaagc | 55 |
| 248 | SLYD 52 | *S. lycopersicoides* | atgcttactctccctgagtg | cacgttcatcaagctctctc | 55 |
| 249 | SLYD 53 | *S. lycopersicoides* | ttaagtggatgtgccggaag | gctagagtacttagaggagc | 53 |
| 250 | SLYD 54 | *S. lycopersicoides* | caaaaaagcaccaccggatc | ttcaacagagctaacgcctc | 55 |
| 251 | SLYD 55 | *S. lycopersicoides* | tacctaatcgtgtgccagtg | accgtagtgcacattctctc | 55 |
| 252 | SLYD 56 | *S. lycopersicoides* | tccatggcatttccttcacc | aaagccagcagagataagcc | 55 |
| 253 | SLYD 57 | *S. lycopersicoides* | aattggccattagcaccacc | gtcgcatttgccaagtcatc | 55 |
| 254 | SLYD 58 | *S. lycopersicoides* | aacttgttggaactacggcc | atacacgtgggacgataagc | 55 |
| 255 | SLYD 59 | *S. lycopersicoides* | cgctagcatttcaactgcag | aaccccagacaagtcttgac | 55 |
| 256 | SLYD 60 | *S. lycopersicoides* | tgatggttctattccccacc | cttaatgtcagcagcagctg | 55 |
| 257 | SLYD 61 | *S. lycopersicoides* | atgtcaagggatgtctgctg | tcacaaagcacgacaacgac | 53 |
| 258 | SLYD 62 | *S. lycopersicoides* | acctgtgtgcaggatgtttg | ccaaggatggtgctatcatg | 55 |
| 259 | SLYD 63 | *S. lycopersicoides* | tggatcaaccttctctaccc | aatgagccaggatctgttgg | 55 |
| 260 | SLYD 64 | *S. lycopersicoides* | tcatccgcttgatactgcag | attgatggtggcagtggttg | 55 |
| 261 | SLYD 65 | *S. lycopersicoides* | acaggaattcactggtcacc | ataggctaccctcgatttgg | 55 |
| 262 | SLYD 66 | *S. lycopersicoides* | attgcttcatgctgcatccc | gaagttgcggcaacagatac | 55 |
| 263 | SLYD 67 | *S. lycopersicoides* | cacctcaaaccctttggttg | tcgagtggaactgaaatggg | 55 |
| 264 | SLYD 68 | *S. lycopersicoides* | ttgctcaggaggatctcaac | agttctcagactatgggtgc | 55 |
| 265 | SLYD 69 | *S. lycopersicoides* | acaaagatcgcctcttggtg | ggcagctactttatcactgg | 55 |
| 266 | SLYD 70 | *S. lycopersicoides* | ctattttagccacgggagag | ctgtgcatctttcctgtgac | 53 |
| 267 | SLYD 71 | *S. lycopersicoides* | catacggcttttccaggttg | tgtggggattctgcattacc | 55 |
| 268 | SLYD 72 | *S. lycopersicoides* | caaggagctgggtaaaaacc | ctgtaccctccaacagaaag | 55 |
| 269 | SLYD 73 | *S. lycopersicoides* | agaagctcctttgccagttg | aacacagactgagaggagac | 55 |
| 270 | SLYD 74 | *S. lycopersicoides* | ccccatgcagttcaagtaac | agtggggtgggattgttttg | 53 |
| 271 | SLYD 75 | *S. lycopersicoides* | cggtaagtcactagaacgag | ctactcctagctagctctac | 55 |
| 272 | SLYD 76 | *S. lycopersicoides* | aaaaacgtgctcctccatcc | actctagaaacactccggag | 55 |
| 273 | SLYD 77 | *S. lycopersicoides* | tgtggtgagaagactgcaag | ctgtgttccacccttttgtc | 55 |
| 274 | SLYD 78 | *S. lycopersicoides* | tccggcgaaattttacaccc | atcttctcgttgcatgcgtc | 55 |
| 275 | SLYD 79 | *S. lycopersicoides* | agcacagctgagagtagtag | caatctgcagtccgtagatc | 55 |
| 276 | SLYD 81 | *S. lycopersicoides* | ttccagcaccaagtgtttcc | agacaggaatgttgcgcatc | 55 |
| 277 | SLYD 82 | *S. lycopersicoides* | ttccacttgtggacgcaaac | ttatgagccaaatgccaccc | 55 |
| 278 | SLYD 83 | *S. lycopersicoides* | accttaccttccagattccc | tgctgtcatccaagtttcag | 55 |
| 279 | SLYD 84 | *S. lycopersicoides* | tacttcacttgtgatccccc | tgcagaaagtttggaggcag | 55 |
| 280 | SLYD 85 | *S. lycopersicoides* | acactactcttctgctctcc | caagtttggggatgttgagg | 55 |
| 281 | SLYD 86 | *S. lycopersicoides* | tatgcacgtacatttggcgc | tgttcccaacatggacaagg | 55 |
| 282 | SLYD 87 | *S. lycopersicoides* | gttatggagttgtgtgctgg | acagcccaaaatctgttgcc | 53 |
| 283 | SLYD 88 | *S. lycopersicoides* | atgttggagccttgccaaag | gggcgttgaaaagatcgaag | 55 |
| 284 | SLYD 89 | *S. lycopersicoides* | ttgaaaagcacctcccatcg | actgatgcctaagttgctcg | 53 |
| 285 | SLYD 90 | *S. lycopersicoides* | atacctgttgctacttccgc | gggaagtcgcaaagtgaatc | 53 |
| 286 | SLYD 91 | *S. lycopersicoides* | tagctatcagctgcttgcag | atgaggtcttctgttgtggc | 53 |
| 287 | SLYD 92 | *S. lycopersicoides* | acatcaaaccccgcagaatc | gtgggatgaaaaagctggag | 55 |
| 288 | SLYD 93 | *S. lycopersicoides* | gacaaagcttggggtacttg | aggtgacctgggtataactg | 55 |
| 289 | SLYD 94 | *S. lycopersicoides* | aggccaagcaacagctattc | taccttcccatacctgagac | 53 |
| 290 | SLYD 95 | *S. lycopersicoides* | aattcatgcaagtgtgcccc | cggatccttcacaagtagtg | 55 |
| 291 | SLYD 96 | *S. lycopersicoides* | gttgtagcgggtaaagaatttg | tactcgaattaccacacgcctc | 53 |
| 292 | SLYD 97 | *S. lycopersicoides* | aatgctcggtctgcaatagc | ggtgaatgcgaaacttcagg | 53 |
| 293 | SLYD 98 | *S. lycopersicoides* | tgctgattcatcgaatggcc | aagagttgaggggcttcaag | 55 |
| 294 | SLYD 99 | *S. lycopersicoides* | tgtctgccttgaggctaaag | agcaggaatctacaggatgg | 55 |
| 295 | SLYD 100 | *S. lycopersicoides* | ttttccagtctccagcgaac | ttcgagtagcactcaactgg | 55 |
| 296 | SLYD 101 | *S. lycopersicoides* | gagatataacccattgcacc | gttgcaacaagagaagggag | 55 |
| 297 | SLYD 102 | *S. lycopersicoides* | atgacatctcgcagacaagg | cttccaaggcacatatctcc | 55 |
| 298 | SLYD 103 | *S. lycopersicoides* | aagaagccagatggtgaacc | atagccttcgtaaagggcag | 55 |
| 299 | SLYD 104 | *S. lycopersicoides* | ataaaagaggaggcctcacc | ccttgaaccacatctgatac | 55 |
| 300 | SLYD 105 | *S. lycopersicoides* | cctctctaatgacgagttgc | gttcagatcatgcccctatg | 55 |
| 301 | SLYD 106 | *S. lycopersicoides* | catggtttatcctgctggac | tgcccgagagagtattctag | 55 |
| 302 | SLYD 107 | *S. lycopersicoides* | atactggtgaacacctcagg | aaatcttcagctcagcctgc | 55 |
| 303 | SLYD 108 | *S. lycopersicoides* | ttgttgttggcactggatcg | gagcatggcatatccttcac | 55 |
| 304 | SLYD 109 | *S. lycopersicoides* | tgggagatcatgagttgctg | actacataggaagcacctgg | 55 |
| 305 | SLYD 110 | *S. lycopersicoides* | ttgaccagcagagaagcttg | aaaaccaggagttcgcaagc | 55 |
| 306 | SLYD 111 | *S. lycopersicoides* | ttactttcccccgtgaactc | actgcagtcgaagatggtag | 55 |
| 307 | SLYD 112 | *S. lycopersicoides* | caggtatccggagaacatag | acaccgatattccttgtggc | 55 |
| 308 | SLYD 113 | *S. lycopersicoides* | cagcttccagaattctgctc | ggatggtatgtaaccgaagc | 55 |
| 309 | SLYD 114 | *S. lycopersicoides* | cctgtattgtattgaacagg | cttaccttgtgttgcctctac | 55 |
| 310 | SLYD 115 | *S. lycopersicoides* | ctcagtgtatctaatgcctg | tcttgcatgaattgaagcgc | 55 |
| 311 | SLYD 116 | *S. lycopersicoides* | actgttcgacccatcagatg | caaggttatcatcccttcgg | 55 |
| 312 | SLYD 117 | *S. lycopersicoides* | tatcgaccaaagtcacggac | caaggttatcatcccttcgg | 55 |
| 313 | SLYD 118 | *S. lycopersicoides* | gaccctatgcagttcaatcc | ccaacatggtgacttttgcc | 55 |
| 314 | SLYD 119 | *S. lycopersicoides* | tgcacctttgggagattcac | acattctatctgcttctccc | 55 |
| 315 | SLYD 120 | *S. lycopersicoides* | ggtcggctataaccaacatc | gaattccctcaccccatttc | 55 |
| 316 | SLYD 121 | *S. lycopersicoides* | ttactgtgcctgtggagaag | tacatcctttggtatggggc | 55 |
| 317 | SLYD 122 | *S. lycopersicoides* | aagcttagcaaaaccgccac | gctcttcagtgcatatacgc | 55 |
| 318 | SLYD 123 | *S. lycopersicoides* | ggatagctgatgtatcaccc | agaagggatagagccaaagc | 55 |
| 319 | SLYD 124 | *S. lycopersicoides* | gccttcaaaaggcggaaaac | acagtggcttgtgatctctc | 55 |
| 320 | SLYD 125 | *S. lycopersicoides* | gattttcaagcggcttgacc | actatgcctccaatggtagc | 55 |
| 321 | SLYD 126 | *S. lycopersicoides* | acttcagctcggtaacactg | ccatcaacaactaccaatgg | 55 |
| 322 | SLYD 127 | *S. lycopersicoides* | gctaaacacagccctgaaac | agagtcacaactactcgtgg | 55 |
| 323 | SLYD 128 | *S. lycopersicoides* | tatcctggtctgaccatgag | tgactggagatgaagcaagc | 55 |
| 324 | SLYD 129 | *S. lycopersicoides* | cacctagtcatctaggttcc | agaagaatcttagcggaggc | 55 |
| 325 | SLYD 130 | *S. lycopersicoides* | aattgttcgtgtgtgggttc | ggatcacagttgaaatcacg | 55 |
| 326 | SLYD 131 | *S. lycopersicoides* | ccattagatgctccatagtc | ccacctatcaaacgaactag | 55 |
| 327 | SLYD 132 | *S. lycopersicoides* | ccatgagaatcagagacttg | gctttcctttttacttgagg | 55 |
| 328 | SLYD 133 | *S. lycopersicoides* | aacacctcaggttcatggag | ctacacactgcctcaatacc | 55 |
| 329 | SLYD 134 | *S. lycopersicoides* | tagtcctctcggtcccagag | accacaactgggattgtggg | 55 |
| 330 | SLYD 135 | *S. lycopersicoides* | tgcattaaaagggtgggttg | ggaacctgaaataggaaagg | 55 |
| 331 | SLYD 136 | *S. lycopersicoides* | agaagggctcaactcttacc | gctaactccaaggcttcaca | 55 |
| 332 | SLYD 137 | *S. lycopersicoides* | gtgatgaggttgatattggg | aagatgtgatttcccaagcc | 53 |
| 333 | SLYD 138 | *S. lycopersicoides* | agaggtatcatcttgatggg | aatacttgtctcgcatgtcc | 53 |
| 334 | SLYD 139 | *S. lycopersicoides* | caaccagtcatgtcggtttc | gagggaggaggaacaattag | 55 |
| 335 | SLYD 140 | *S. lycopersicoides* | tatagatccgggagttcgac | ccctatttccacctttgtcc | 55 |
| 336 | SLYD 141 | *S. lycopersicoides* | gcaatcacctagttcccaac | agctgcactagttctgcttc | 55 |
| 337 | SLYD 142 | *S. lycopersicoides* | catcagtgtcagcaaccatg | atctcgtcctgcagattcct | 55 |
| 338 | SLYD 143 | *S. lycopersicoides* | tttctccattggcatccatc | gttagagtcagatgtgatgc | 55 |
| 339 | SLYD 144 | *S. lycopersicoides* | acttcttgggatgagttggc | ttgagatcttccctgctctg | 55 |
| 340 | SLYD 145 | *S. lycopersicoides* | tgggttgcagctaaaacctg | tggagatggatttgtggagg | 53 |
| 341 | SLYD 146 | *S. lycopersicoides* | gcaccaacgcaccttgtgga | ccctgaaacttgaaaggctc | 55 |
| 342 | SLYD 147 | *S. lycopersicoides* | ctcccttcaatgagctggag | ctcaatcagtctctgttagg | 55 |
| 343 | SLYD 148 | *S. lycopersicoides* | ttgtccaccttggactttgc | cctgagaaatggagcatttc | 55 |
| 344 | SLYD 149 | *S. lycopersicoides* | tatgatgtgtagcaccagag | ttgtatgggaggaatgggcc | 55 |
| 345 | SLYD 150 | *S. lycopersicoides* | aattgcaggcctttgcgatg | caacgtcaagtgaagatcgg | 55 |

*^a^S. lycopersicoides*-specific SSRs

^b^*S. lycopersicoides*-specific indel markers

^c^annealing temperature
