# Supplementary figures and images for: Development of a PCR-based, genetic marker resource for the tomato-like nightshade relative, Solanum lycopersicoides using whole genome sequence analysis
Source: PLoS One. 2020 Nov 23;15(11):e0242882. doi: 10.1371/journal.pone.0242882 (PMC7682897; doi:10.1371/journal.pone.0242882)

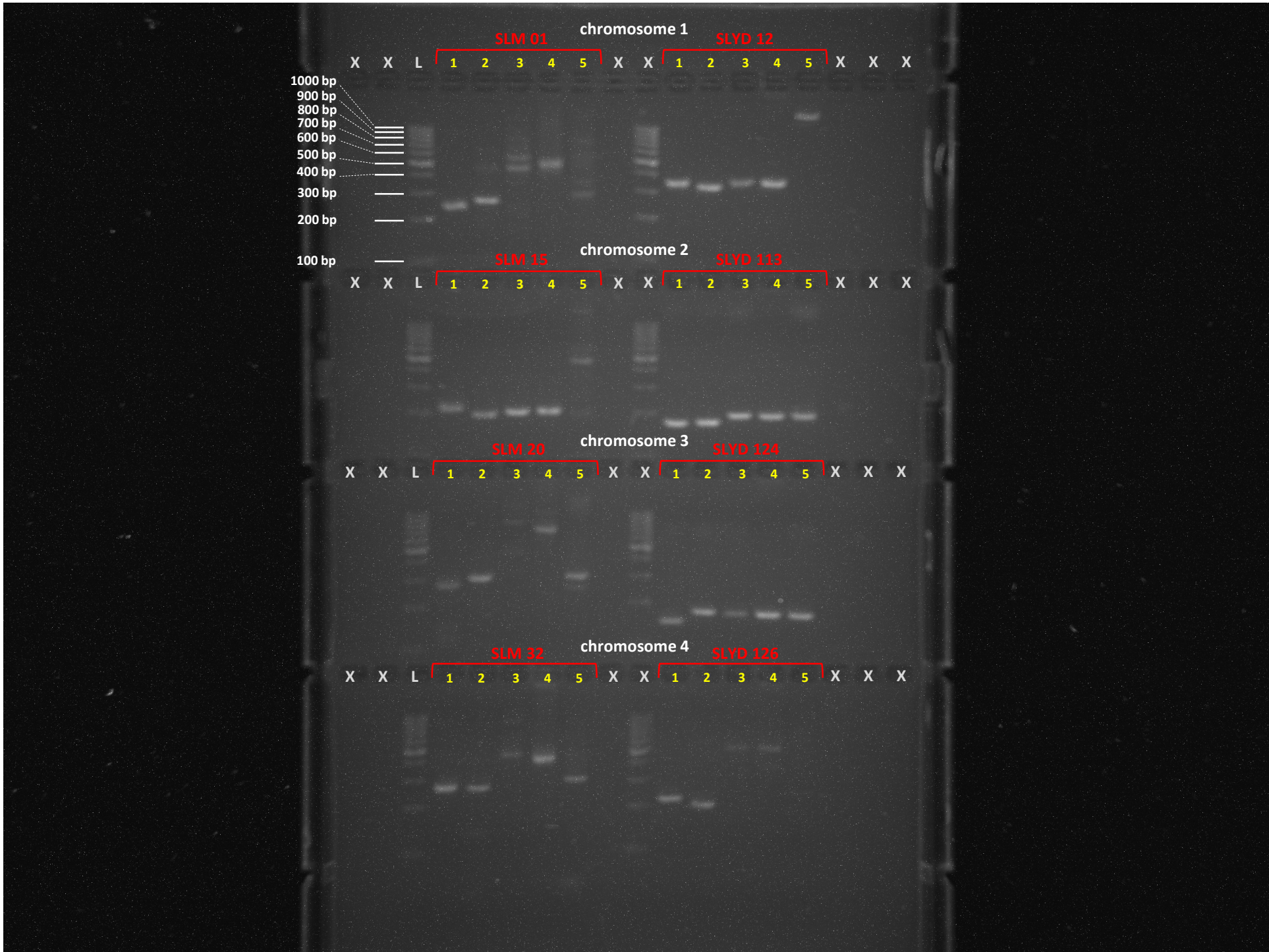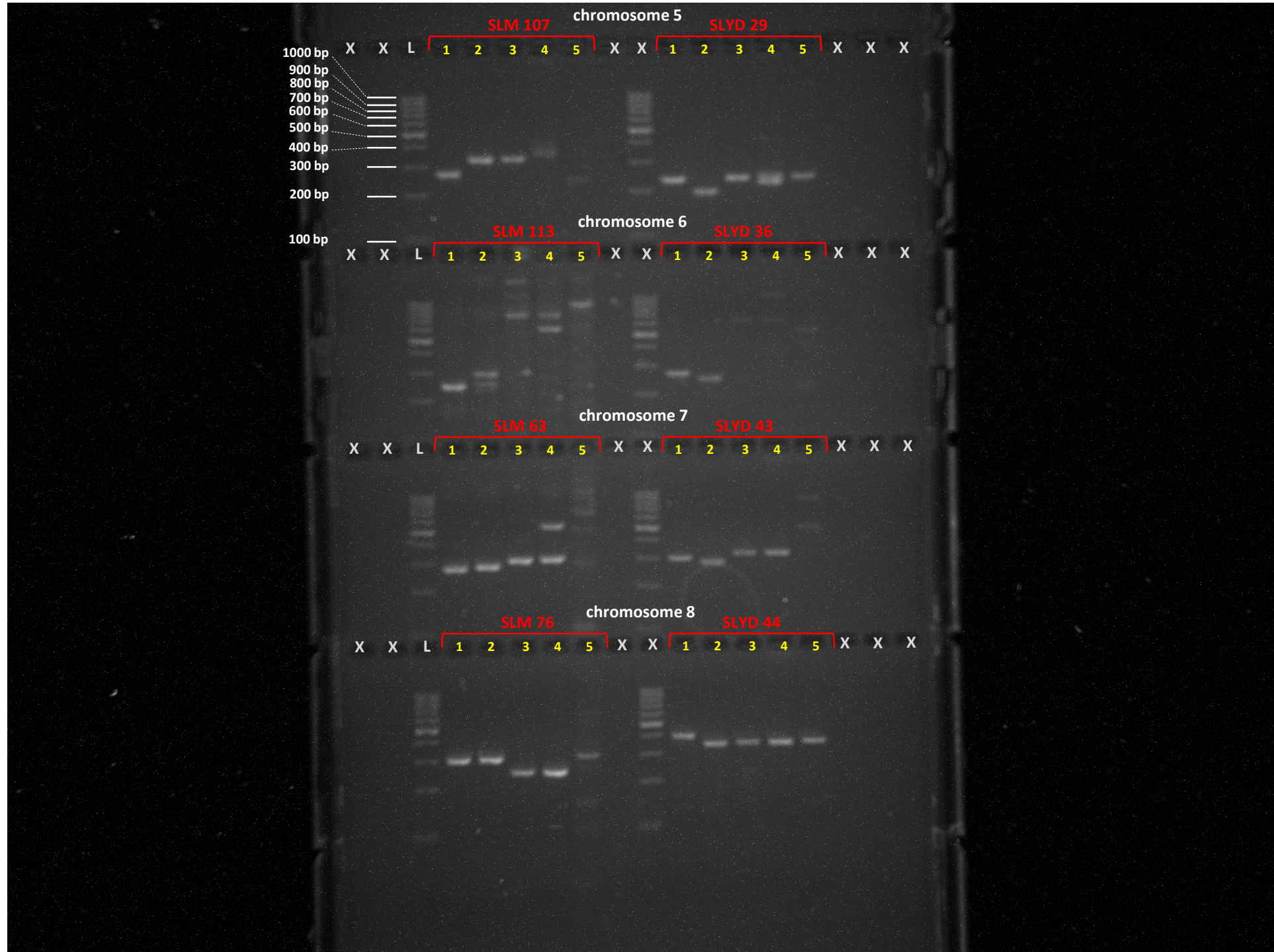

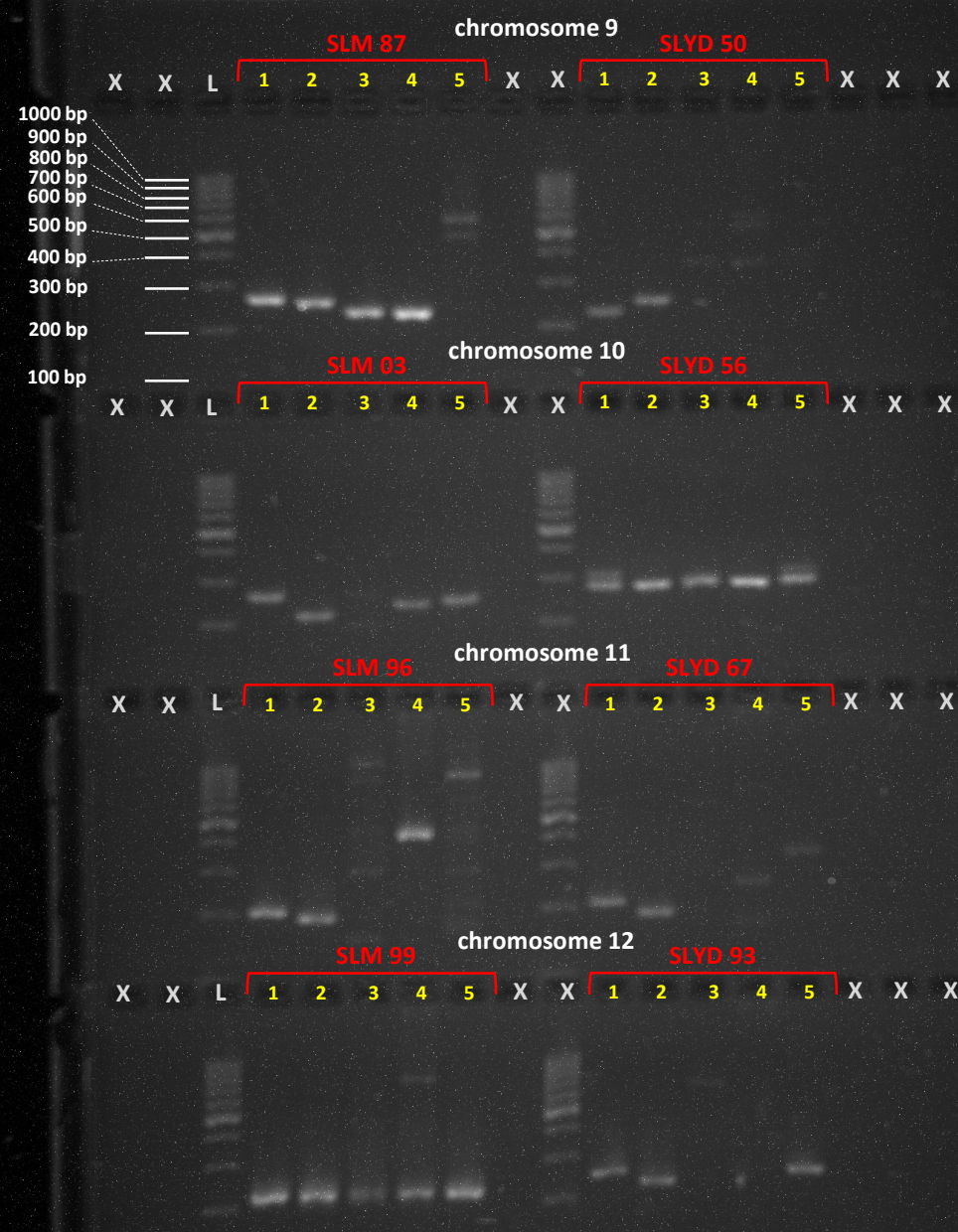

Supplement: S1 Raw images — One SSR and one indel marker for each chromosome were used to amplify targets in tomato, silverleaf nightshade, eggplant and pepper. SLM = SSR marker, SLYD = indel marker, 1 = S. lycopersicoides, 2 = tomato, 3 = silverleaf nightshade, 4 = eggplant, 5 = pepper, L = 100 bp-ladder. Lanes marked in X were not used to generate Fig 4. (PDF) [file pone.0242882.s003.pdf]
